# Post-fertilization expression of *FLOWERING LOCUS T* suppresses reproductive reversion

**DOI:** 10.3389/fpls.2014.00164

**Published:** 2014-04-30

**Authors:** Liangyu Liu, Sara Farrona, Sonja Klemme, Franziska K. Turck

**Affiliations:** ^1^Max Planck Institute for Plant Breeding Research, Carl von Linné Weg 10Cologne, Germany; ^2^Key Laboratory of Tropical Forest Ecology, Xishuangbanna Tropical Botanical Garden, Chinese Academy of Sciences, KunmingYunnan, China; ^3^University of Chinese Academy of SciencesBeijing, China

**Keywords:** flowering, *FLOWERING LOCUS T*, floral reversion

## Abstract

*FLOWERING LOCUS T* (*FT*) encodes a systemic signal communicating the perception of long day photoperiod from leaves to the shoot apex to induce the floral transition. Transient expression of *FT* in the phloem companion cells of rosette leaves for one to several days was previously shown to be sufficient to commit plants to flowering. Here we show that partial commitment results in pleiotropic inflorescence meristem reversion phenotypes. *FT* expression is much stronger in organs formed after the floral transition such as cauline leaves, sepals, and developing siliques. We show that expression of *FT* and its paralog *TWIN SISTER OF FT* (*TSF*) after the floral transition plays a role in inflorescence meristem stabilization even if plants flower very late in development. CONSTANS (CO), the major activator of *FT*, is not required to prevent late reproductive reversion. The requirement for *FT* is temporal since reproductive reversion to a vegetative state occurs only in recently formed inflorescence meristems. Unlike for the expression of *FT* in leaves, neither the distal putative *FT* enhancer nor long-day photoperiod is required for *FT* expression in developing siliques. Expression of *FT* in developing siliques and their supporting stems is sufficient to stabilize flowering during the sensitive developmental window indicating that fruit generated FT participates in inflorescence stabilization.

## Introduction

In many plant species, *FLOWERING LOCUS T* (*FT*)-like genes play a critical role in the photoperiod dependent timing of the transition from the vegetative to the reproductive stage (Ballerini and Kramer, 2011). In the model plant *Arabidopsis thaliana*, *FT* integrates environmental and developmental variables at the level of its transcriptional regulation (Andres and Coupland, 2012). As part of florigen, FT protein produced in the leaves migrates to the shoot apical meristem where it triggers the reprogramming of regulatory networks resulting in a change from vegetative to reproductive growth (Corbesier et al., 2007). Prior to the floral transition, *FT* expression is restricted to the leaves and only occurs if days are longer than the critical day length (Suarez-Lopez et al., 2001; Yanovsky and Kay, 2002; Adrian et al., 2010). Photoperiod control of *FT* is dependent on CONSTANS (CO), which acts as direct transcriptional activator presumably in a complex involving NF-Y transcription factors (Wenkel et al., 2006; Kumimoto et al., 2008, 2010; Tiwari et al., 2010). If days are longer than the critical day length, CO protein is stabilized by light and thus capable of promoting *FT* expression (Valverde et al., 2004). In the absence of light, CO is rapidly degraded in the dark and unable to promote *FT* expression. Apart from photoperiod, other external and internal cues participate in *FT* regulation. For example, an increase in ambient temperature overrules *FT*'s dependency on photoperiod and CO by affecting chromatin accessibility (Kumar et al., 2012). In biennial Arabidopsis plants, high levels of the transcription factor FLOWERING LOCUS C (FLC) prior vernalization prevent the activation of *FT* in long-day photoperiod (Hepworth et al., 2002; Michaels et al., 2005). FLC directly binds to putative regulatory regions in the first intron of *FT* (Searle et al., 2006). The effect of FLC is partially dependent on the presence of SHORT VEGETATIVE PHASE (SVP), with which it may form a complex (Hartmann et al., 2000; Li et al., 2008). SVP preferentially binds to regions in the *FT* promoter containing several putative CArG boxes. The effect of SVP repression on *FT* is particularly visible in young plants as well as in cold ambient temperature (Lee et al., 2007). Plant age participates in *FT* regulation via the *microRNA 156* (*miR156*) pathway (Mathieu et al., 2009).

Recently, more pleiotropic roles of *FT* and *FT*-like genes have been reported (Pin and Nilsson, 2012). During the analysis of multi-parent recombinant inbred lines in Arabidopsis, *FT* was identified as quantitative trait locus (QTL) implicated in the formation of axillary meristems after. but not before, the floral transition (Huang et al., 2013). *FT* and its closest paralog *TWIN SISTER OF FT* (*TSF*) were shown to play a role in side shoot outgrowth with *ft* and *tsf* single mutants showing a reduced elongation rate of side shoots in long days (LD) and short days (SD), respectively (Hiraoka et al., 2013). Double *ft;tsf* mutants showed an enhanced reduction of side shoot elongation in both photoperiods. In addition, *FT* expression in stomata was shown to result in stomata opening, indicating a participation of *FT* in transpiration control (Kinoshita et al., 2011). In potato, some *FT*-like genes were shown to be involved in tuber initiation in response to long days, whereas other paralogs were regulating flowering in short days (Navarro et al., 2011). In poplar, high expression of *FT*-like genes causes early flowering and thus overcomes the juvenility phase that usually prevents the floral transition in the first years of development (Bohlenius et al., 2006; Hsu et al., 2006, 2011). However, expressed at lower levels, functional divergence of poplar *FT1* and *FT2* paralogs becomes obvious. Poplar *FT1*, which is induced by prolonged cold in various tissues including buds and leaves, triggers the onset of reproductive bud formation, whereas the long-day induced poplar *FT2*, which expresses in the leaves, is much less effective in this process. Moderate expression of *FT2* prevents growth cessation, which is usually triggered in short days or after stress perception in natural conditions but is not sufficient to trigger reproductive bud formation. Thus the role of *FT2* is to maintain vegetative growth during the growing season (Hsu et al., 2011).

Interestingly, *FT* in Arabidopsis is expressed at much higher levels after the floral transition, notably in cauline leaves, sepals, petals and developing siliques (Schmid et al., 2005; Adrian et al., 2010), and the expression in developing siliques is independent of photoperiod (Hiraoka et al., 2013). Here we set out to elucidate if *FT* expression in developing fruits fulfills a biological function or rather reflects a non-functional relaxation in the tight repressive control of transcription that is necessary to prevent precocious flowering earlier in development. We show that photoperiod-independent expression of *FT* in developing fruits plays a role in inflorescence maintenance.

## Results

### Several days of induction of FT expression are required for full floral commitment of arabidopsis plants to flower

**Figure 1:**
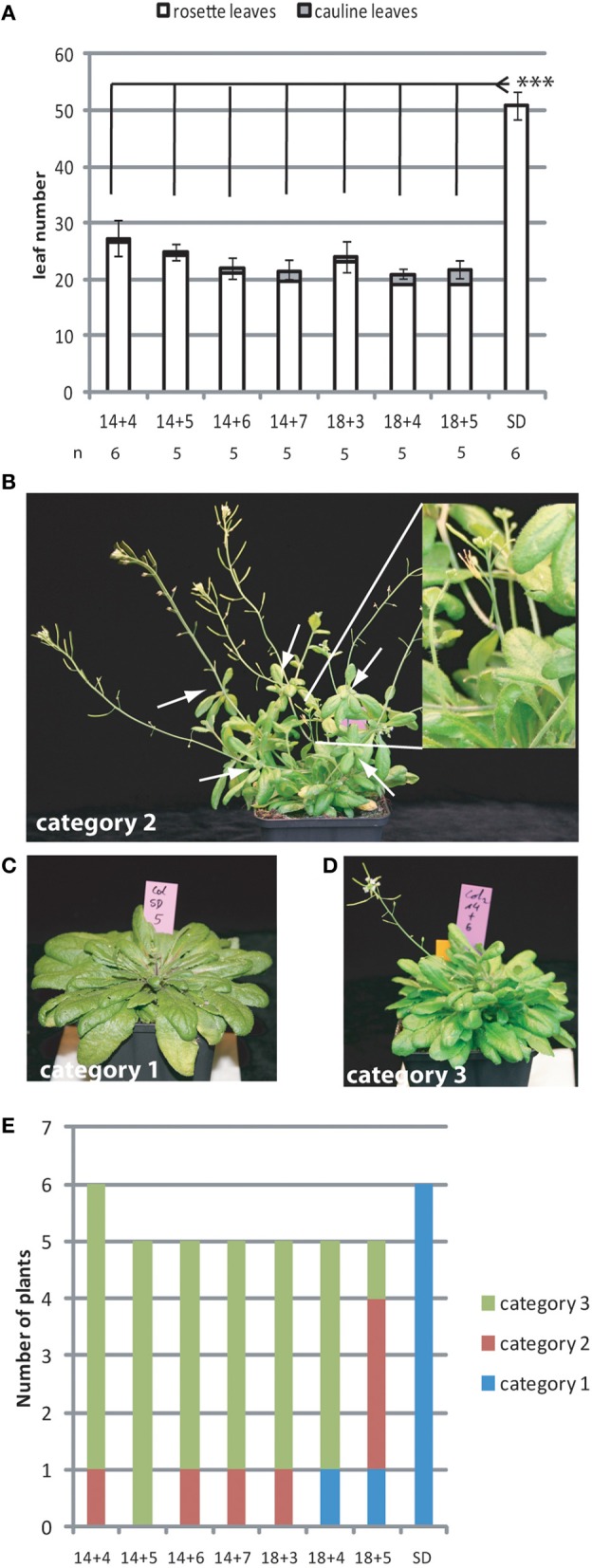
**Several ESDs are required to fully commit the shoot apical meristem to flowering**. Col plants grown in SD for 14 or 18 days were transiently shifted to an increasing number of days in ESD as indicated. **(A)** Flowering was determined by counting rosette and cauline leaves of the main inflorescence. In the case of SD grown plants only rosette leaves were counted. Numbers of total leaves are shown as the mean ± SE. Statistically significant differences between treatments was assessed by One-Way ANOVA followed by Tukey Honest Significant Differences (^***^p < 0.01). **(B–D)** Plants typical for phenotypic categories 1–3, respectively. Categories are: “1: as wild-type,” “1: reduced apical dominance and aerial rosettes at main shoot,” “2: main shoot arrested, aerial rosettes on side shoots at the rosette” White arrows indicate aerial rosettes, the inlet shows an arrested main shoot without apical dominance. **(E)** Quantification of the number of plants per category observed for different photoperiod treatments as indicated.

Previous studies reported that 3 days in LD growth conditions during which *FT* is expressed are sufficient to induce early flowering in the Arabidopsis accession L*er* whereas Col plants require 5 LDs for full induction of early flowering (Corbesier et al., 2007; Torti et al., 2012). We wanted to quantify more precisely how *FT* expression contributed to the commitment of the plants to flowering. We grew Arabidopsis plants of the accession Col-0 in 18 SD before shifting them transiently for an increasing number of days to extended short day (ESD) conditions and back to SD. ESD growth conditions provide 8 h of full light supplemented by 8 h of low intensity light sufficient to trigger long day (LD) light responses, such as *FT* induction, while minimizing the differences in growth rate observed between plants grown in SD and LD conditions. As expected, plants showed accelerated flowering after three ESDs as compared to control plants grown in SD conditions (Figure 1A).

Plants flowering after experiencing few ESDs showed partial transition/reversion phenotypes. We grouped all shifted plants into three progressive phenotypic categories, which were “1: as wild-type with a main shoot showing clear apical dominance and cauline leaves with a different shape than rosette leaves,” “2: shortened main shoot without apical dominance and with aerial rosettes,” “3: main shoot fully arrested with less than 1 cm bloting or not detectable, frequent aerial rosettes on side shoots formed at the axils of rosette leaves” (Figures 1B–D). The severity of the phenotype was inversely correlated with the number of ESDs the plants had experienced (Figure 1E) indicating that maintained *FT* expression participates in preventing reversion of the inflorescence meristem to a more vegetative or arrested state. In addition, plants that were older at the start of the ESD treatment showed less severe phenotypes than plants treated at a younger age (Figure 1E).

### Transient reproductive reversion is observed in *ft* mutant plants

**Figure 2:**
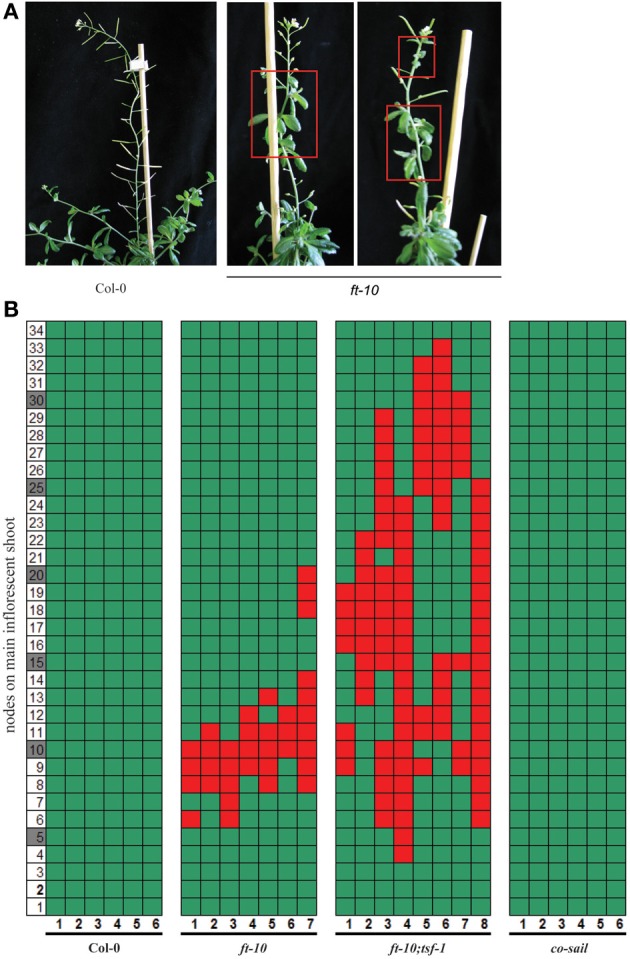
**Reversion phenotype of photoperiod pathway mutants in SDs**. Plants were grown under SD conditions. **(A)** Plants typical for flowering phenotype of Col-0 wildtype and *ft-10* mutant **(B)** Side shoots on the main inflorescence were counted from the first flower (1) toward the top (until node 34). Flowers are indicated by green, cauline leaves by red squares, the number of plants assessed is indicated on the x-axis.

The hypothesis that maintained *FT* expression is required to prevent reversion of the inflorescence is challenged by the fact that reversion has not previously been reported for plants grown either in SD growth conditions or carrying non-functional *FT* alleles. We assessed whether reversion of flowering could have been overlooked in plants that flower late in development. Although we never observed reversion in Col-0 plants grown in SDs, partial reproductive reversion was commonly observed in *ft-10* mutants (Figures 2A,B). This reproductive reversion phenotype was different from that observed in younger plants induced by a suboptimal length of ESDs, since bolting rate or apical dominance of the main shoot were not affected (Figure 1). Instead, reversion was observed in flowering shoots early after the formation of the first true flowers. The inflorescence meristem reverted to form vegetative side branches with rosette-like cauline leaves (Figures 2A,B). Reversion was transient, affecting between 3 and 10 nodes before the formation of flowers was resumed. Furthermore, reversion was not observed in *co* mutants but was enhanced in *ft-10;tsf-1* double compared to *ft-10* single mutants, which also showed a tendency to go through repeated cycles of reversion (Figures 2A,B). Taken together, the results indicate that the reversion phenotype is independent of CO but dependent on the expression of the two FT-like genes *FT* and *TSF*.

### *FT* expression in developing fruits is not dependent on photoperiod and the distal *FT* enhancer *Block C*

**Figure 3:**
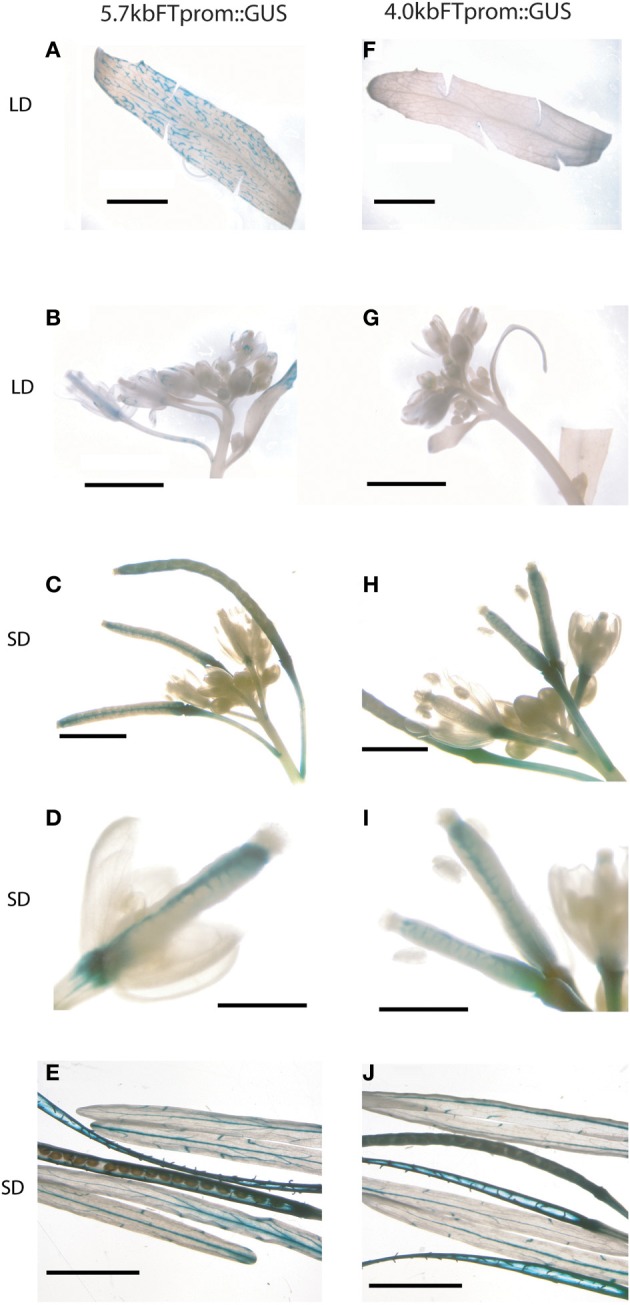
***GUS* driven by *4 kb FT* promoter in siliques**. Histochemical GUS assay of **A–E** *5.7 kbFTp::GUS* and **F–H** *4 kbFTp::GUS* plants in Col-0 background harvested after bolting. Samples were cauline leaves in LDs with cut margins to allow better infiltration of staining solution **(A,F)**, inflorescences grown in LD **(B,G)**, and SDs **(C,H)**, young siliques **(D,I)** and mature siliques grown in SDs **(E,J)**. Scale bars 5 mm, except **(D,I)**: 0.5 mm.

Before the reproductive transition,*FT* is expressed in the phloem companion cells of the minor leaf veins if CO protein is stabilized in response to LD photoperiod (Valverde et al., 2004). After the transition, the expression level of *FT* dramatically increases in the cauline leaf veins in long days (Figure 3A and Supplemental Figure 1). *FT* expression is also detected along the vascular bundles of sepals, petals, the funiculi and septum of developing siliques and in the vasculature of floral stems during silique maturation as indicated by a *GUS* reporter gene driven by the full-length 5.7kb *FT* promoter (Figures 3B–E). Expression of *FT* in developing siliques and supporting stems under the control of this promoter is independent of LD photoperiod (Figures 3C–E). This is similar to data shown by Hiraoka et al., who used a genomic *FT:GUS* fusion that included all intronic regions and a slightly longer *FT* promoter (Hiraoka et al., 2013). We have previously shown that induction of *FT* by LD photoperiod requires the presence of a putative distal enhancer region located between 5.7 and 4.0 kb of the transcription start site (Adrian et al., 2010). While expression of *FT* in all leaf tissues before fertilization was dependent on the presence of the distal regulatory regions (Figures 3F,G) its expression in the funiculi, the septum of developing siliques and the supporting stem did not require the presence of this putative enhancer indicated by the detection of GUS reporter gene expression under the control of a 4.0 kb *FT* promoter (Figures 3H–J).

Taken together, the data indicate that distal regulatory regions are important to express *FT* highly in cauline leaves whereas *FT* utilizes a different regulatory network for activation in developing siliques where the expression is also photoperiod independent.

### Reversion of the inflorescence meristem is prevented by expression of *FT* in developing siliques

**Figure 4:**
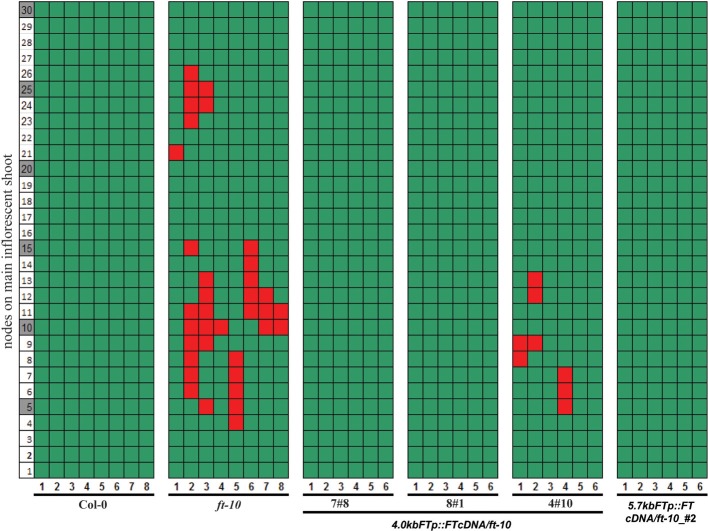
**Reversion of *ft* mutants is abolished by expressing *FT* under the control of a 4.0 kb *FT* promoter**. Reversion phenotype of*ft-10* plants carrying constructs to drive *FT* cDNA by *5.7* and *4.0 kbFTp* were measured. Three independent transgenic lines are shown for *4.0 kbFTp* construct. Col-0, *ft-10*, and *co-sail* were also measured. The nodes of siliques on the major influence shoot are marked as green boxes, and the reverted vegetative nodes are shown as red boxes.

Reproductive reversion in *ft-10* mutants was observed in SD growth conditions when expression of *FT* is mainly restricted to the developing siliques and their stems indicating that expression in these organs after fertilization may participate in maintaining the recently committed inflorescence meristem. We made use of the observation that the *4kbFTprom::GUS* transgene was exclusively expressed in these tissues to test the functional relevance of *FT* expression in these organs. Transgenic plants that expressed *FT* cDNA under the control of the 4 kb *FT* promoter in the *ft-10* background showed no inflorescence reversion in two out of three transgenic lines whereas a third line showed a reduced reversion phenotype (Figure 4). This was not different from the rescue of the reversion phenotype observed when *FT* cDNA was expressed under the control of the full length 5.7 kb *FT* promoter (Figure 4). The floral reversion type was not correlated to slight differences in flowering time that were observed between the transgenic plants and the parental *ft-10* mutant in SD growth conditions (Supplemental Figure 2).

Taken together, the data indicate that photoperiod independent expression of *FT* in developing siliques and their supporting stem is functional and participates in maintaining the commitment of the inflorescence meristem.

## Discussion

We addressed the question whether expression of *FT* plays a functional role in Arabidopsis development after the plants have transitioned to flowering. The interest in solving this question was ignited by the previous observation that *FT* levels are much higher in organs formed after the floral transition than in rosette leaves (Supplemental Figure 1). Recently, it has been reported that *FT* and the related *TSF* play a role in controlling the elongation rate of side shoots developing from subtending cauline leaves and that FT protein moves from cauline leaves to axillary meristems (Hiraoka et al., 2013; Niwa et al., 2013). Our data suggest that elevated *FT* levels after the transition to flowering help stabilizing the recently formed inflorescence meristem, which seems particularly required if plants flower early in development.

Reversion of the inflorescence meristem in young plants by insufficiently persistent photoperiod signals can lead to dramatic phenotypic effects the most severe being the full arrest of the main apical meristem (Figure 1). It is currently unclear if this is an accidental consequence of the repeated reorientation of meristem identity or a deliberate developmental program. It can be argued that plants growing in natural conditions in autumn may profit from inflorescence arrest if this permits them to resume vegetative growth until the next spring instead of flowering in late autumn at an early developmental stage.

Inflorescence reversion after the formation of flowers was previously reported for plants carrying mutations in both, *SUPRESSOR OF CONSTANS 1 (SOC1)* and *FRUITFUL (FUL)* (Melzer et al., 2008). *SOC1* and *FUL* act with partial redundancy to accelerate flowering in LD (Melzer et al., 2008) and attenuate the effect of an ectopic overexpression of *FT* (Melzer et al., 2008; Torti et al., 2012). Inflorescence reversion observed in *soc1;ful* double mutants is suppressed by adding an additional mutation in the gene encoding the floral repressor SHORT VEGETATIVE PHASE (SVP) (Torti et al., 2012). Thus it is likely that the expression of *FT* in developing fruits and their supporting stems serves to maintain the expression of *SOC1*, *FUL*, and other unknown SVP target genes at the inflorescence meristem.

In wild type Col-0 plants grown in SD combined expression of *FT* and *TSF* prevents floral reversion after the formation of the first flowers. Expression of *FT* in the vascular bundles of developing siliques and the supporting stems is sufficient to prevent reversion of newly committed inflorescence meristems. Expression of *FT* in these tissues is independent of photoperiod and does not require the distal regulatory region that we previously showed was required for *FT* activation by CO in rosette leaves (Adrian et al., 2010). Apparently; in these particular tissues *FT* can make use of a distinct set of transcriptional activators to promote expression. These activators may not be expressed or activated in other organs. However, it is also possible that the *FT* locus itself becomes more permissive for transcription activation in developing siliques. Interestingly, the 4 kb *FT* promoter, which is sufficient for driving expression in developing siliques but not in other tissues also activates *FT* ectopically in SD in mutants affecting the chromatin-dependent Polycomb Group (PcG) pathway such as *like-heterochromatin protein 1* (*lhp1*) and *curly leaf* (*clf*) (Adrian et al., 2010; and Supplemental Figure 3). Thus, a reduction in PcG-mediated repression of *FT* could explain photoperiod independent expression in developing siliques and their supporting stems.

Feed-back from developing fruits toward programming of developing meristems is also documented in other plant species, such as fruit trees belonging to the Rosaceae family. Many apple landraces show biennial flowering (alternate bearing) because the ripening fruits inhibit the transition of newly forming meristems to an inflorescent state (Goldschmidt, 2013). However, the molecular mechanisms explaining this developmental program are not understood and it is unclear if *FT* related genes are implicated in the process.

A delay of the reproductive phase has been associated with high performance of cereal crops grown in temperate climates that are not threatened by seasonal water shortage (Jung and Muller, 2009). Given their common potential to promote flowering in response to photoperiod, mutations in *FT*-like genes are likely to delay flowering in many species and thus may represent good targets for breeding. However, more pleiotropic roles of *FT* and related genes after the floral transition suggest that loss of *FT* function could have deleterious effects on overall seed yield. Our study shows that *FT* is important to stabilize the inflorescence meristem and recently it was reported that *FT* expression promotes side shoot formation at the axils of cauline leaves (Huang et al., 2013) as well as the rate of side shoot elongation (Hiraoka et al., 2013). Future studies should elucidate whether the pleiotropic roles *FT* plays after the floral transition are as universal as its involvement in the promotion of flowering.

## Methods

### Plant growth

SD conditions included 8 h of cool-white fluorescent light (8.5 Wm^−2^), followed by 16 h of darkness. Extended SD conditions included 8 h of cool-white fluorescent light, followed by 8 h of low-fluence rate incandescent light (0.2 Wm^−2^), followed by 8 h of darkness. LD conditions included 16 h of cool-white fluorescent light followed by 8 h of darkness. Seeds were sowed on soil and stratified at 4°C for 3 days. Soil trays were transferred to LD or SD growth conditions as indicated. For the shift experiment, plants grown in SDs for 14 or 18 days were transiently shifted to ESDs for 3–7 days and shifted back to SDs until setting seeds. As measure of flowering time, the total rosette, and cauline leaves on the main inflorescence shoot were counted. Plants were grown at 22°C for SD-ESD-SD shift experiments and at 20°C for inflorescence reversion experiments.

### Phenotyping inflorescence reversion

For plants shifted temporarily from SD to ESD conditions, reversion was assessed by grouping plants into three phenotypic categories which were “1: as wild-type with a main shoot showing clear apical dominance and cauline leaves with a different shape than rosette leaves,” “2: shortened main shoot without apical dominance and with aerial rosettes,” “3: main shoot fully arrested with less than 1 cm bloting or not detectable, frequent aerial rosettes on side shoots formed at the axils of rosette leaves.” Reversion for plants grown in SD was assessed on the main inflorescence shoot when plants had initiated inflorescences on all side shoots.

### GUS histochemical staining

Cauline leaves, flowers, and siliques were collected from 2-month-old LD or 3-month-old SD grown plants carrying either a 5.7 or 4.0 kb *FT* promoter GUS constructs as indicated. Samples were incubated in 90% Acetone on ice for 30 min, rinsed with 50 mM sodium phosphate buffer and incubated for 24–36 h at 37°C in GUS staining solution (0.5 mg/ml X-Gluc, 50 mM sodium phosphate buffer, 0.5 mM potassium ferrocyanide, 0.5 mM potassium ferricyanide, 0.1% Triton X-100). To allow better penetration of the staining solution, cauline leaves were cut at the edges. After incubation, samples were washed repeatedly with 50 mM sodium phosphate buffer for 30 min and 70% ethanol until leaves turned white. GUS staining was visualized and photographed under a stereomicroscope (Leica).

### Transgenic plants and mutants

*5.7 kb-FTpro::GUS/FTcDNA;ft-10* and *4.0 kb-FTpro::GUS/FTcDNA;ft-10* transgenic plants have been described previously (Adrian et al., 2010). Mutants *ft-10* (GK-290E08), *tsf-1*(SALK 087522), and *co-10* (SAIL_24_H04) are caused by T-DNA insertions in the Colombia-0 ecotype and have been described previously (Michaels et al., 2005; Yoo et al., 2005; Laubinger et al., 2006).

### Conflict of interest statement

The authors declare that the research was conducted in the absence of any commercial or financial relationships that could be construed as a potential conflict of interest.

## References

[B1] Adrian J. Farrona S. Reimer J. J. Albani M. C. Coupland G. Turck F. (2010). cis-Regulatory elements and chromatin state coordinately control temporal and spatial expression of *FLOWERING LOCUS T* in Arabidopsis. Plant Cell 22, 1425–1440 10.1105/tpc.110.074682 20472817 PMC2899882

[B2] Andres F. Coupland G. (2012). The genetic basis of flowering responses to seasonal cues. Nat. Rev. Genet. 13, 627–639 10.1038/nrg3291 22898651

[B3] Ballerini E. S. Kramer E. M. (2011). In the light of evolution: a reevaluation of conservation in the CO-FT regulon and its role in photoperiodic regulation of flowering time. Front. Plant Sci. 2:81 10.3389/fpls.2011.00081 22639612 PMC3355682

[B4] Bohlenius H. Huang T. Charbonnel-Campaa L. Brunner A. M. Jansson S. Strauss S. H. (2006). CO/FT regulatory module controls timing of flowering and seasonal growth cessation in trees. Science 312, 1040–1043 10.1126/science.1126038 16675663

[B5] Corbesier L. Vincent C. Jang S. Fornara F. Fan Q. Searle I. (2007). FT protein movement contributes to long-distance signaling in floral induction of Arabidopsis. Science 316, 1030–1033 10.1126/science.1141752 17446353

[B6] Goldschmidt E. E. (2013). The evolution of fruit tree productivity: a review. Econ. Bot. 67, 51–62 10.1007/s12231-012-9219-y 23538880 PMC3606516

[B7] Hartmann U. Hohmann S. Nettesheim K. Wisman E. Saedler H. Huijser P. (2000). Molecular cloning of SVP: a negative regulator of the floral transition in Arabidopsis. Plant J. 21, 351–360 10.1046/j.1365-313x.2000.00682.x 10758486

[B8] Hepworth S. R. Valverde F. Ravenscroft D. Mouradov A. Coupland G. (2002). Antagonistic regulation of flowering-time gene SOC1 by CONSTANS and FLC via separate promoter motifs. EMBO J. 21, 4327–4337 10.1093/emboj/cdf432 12169635 PMC126170

[B9] Hiraoka K. Yamaguchi A. Abe M. Araki T. (2013). The florigen genes FT and TSF modulate lateral shoot outgrowth in *Arabidopsis thaliana*. Plant Cell Physiol. 54, 352–368 10.1093/pcp/pcs168 23220822

[B10] Hsu C. Y. Adams J. P. Kim H. No K. Ma C. Strauss S. H. (2011). *FLOWERING LOCUS T* duplication coordinates reproductive and vegetative growth in perennial poplar. Proc. Natl. Acad. Sci. U.S.A. 108, 10756–10761 10.1073/pnas.1104713108 21653885 PMC3127867

[B11] Hsu C. Y. Liu Y. Luthe D. S. Yuceer C. (2006). Poplar FT2 shortens the juvenile phase and promotes seasonal flowering. Plant Cell 18, 1846–1861 10.1105/tpc.106.041038 16844908 PMC1533980

[B12] Huang X. Ding J. Effgen S. Turck F. Koornneef M. (2013). Multiple loci and genetic interactions involving flowering time genes regulate stem branching among natural variants of Arabidopsis. New Phytol. 199, 843–857 10.1111/nph.12306 23668187

[B13] Jung C. Muller A. E. (2009). Flowering time control and applications in plant breeding. Trends Plant Sci. 14, 563–573 10.1016/j.tplants.2009.07.005 19716745

[B14] Kinoshita T. Ono N. Hayashi Y. Morimoto S. Nakamura S. Soda M. (2011). *FLOWERING LOCUS T* regulates stomatal opening. Curr. Biol. 21, 1232–1238 10.1016/j.cub.2011.06.025 21737277

[B15] Kumar S. V. Lucyshyn D. Jaeger K. E. Alos E. Alvey E. Harberd N. P. (2012). Transcription factor PIF4 controls the thermosensory activation of flowering. Nature 484, 242–245 10.1038/nature10928 22437497 PMC4972390

[B16] Kumimoto R. W. Adam L. Hymus G. J. Repetti P. P. Reuber T. L. Marion C. M. (2008). The Nuclear Factor Y subunits NF-YB2 and NF-YB3 play additive roles in the promotion of flowering by inductive long-day photoperiods in Arabidopsis. Planta 228, 709–723 10.1007/s00425-008-0773-6 18600346

[B17] Kumimoto R. W. Zhang Y. Siefers N. Holt B. F. 3rd. (2010). NF-YC3, NF-YC4 and NF-YC9 are required for CONSTANS-mediated, photoperiod-dependent flowering in *Arabidopsis thaliana*. Plant J. 63, 379–391 10.1111/j.1365-313X.2010.04247.x 20487380

[B18] Laubinger S. Marchal V. Le Gourrierec J. Wenkel S. Adrian J. Jang S. (2006). Arabidopsis SPA proteins regulate photoperiodic flowering and interact with the floral inducer CONSTANS to regulate its stability. Development 133, 3213–3222 10.1242/dev.02481 16854975

[B19] Lee J. H. Yoo S. J. Park S. H. Hwang I. Lee J. S. Ahn J. H. (2007). Role of SVP in the control of flowering time by ambient temperature in Arabidopsis. Genes Dev. 21, 397–402 10.1101/gad.1518407 17322399 PMC1804328

[B20] Li D. Liu C. Shen L. Wu Y. Chen H. Robertson M. (2008). A repressor complex governs the integration of flowering signals in Arabidopsis. Dev. Cell 15, 110–120 10.1016/j.devcel.2008.05.002 18606145

[B21] Mathieu J. Yant L. J. Murdter F. Kuttner F. Schmid M. (2009). Repression of flowering by the miR172 target SMZ. PLoS Biol. 7:e1000148 10.1371/journal.pbio.1000148 19582143 PMC2701598

[B22] Melzer S. Lens F. Gennen J. Vanneste S. Rohde A. Beeckman T. (2008). Flowering-time genes modulate meristem determinacy and growth form in *Arabidopsis thaliana*. Nat. Genet. 40, 1489–1492 10.1038/ng.253 18997783

[B23] Michaels S. D. Himelblau E. Kim S. Y. Schomburg F. M. Amasino R. M. (2005). Integration of flowering signals in winter-annual Arabidopsis. Plant Physiol. 137, 149–156 10.1104/pp.104.052811 15618421 PMC548846

[B24] Navarro C. Abelenda J. A. Cruz-Oro E. Cuellar C. A. Tamaki S. Silva J. (2011). Control of flowering and storage organ formation in potato by *FLOWERING LOCUS T*. Nature 478, 119–122 10.1038/nature10431 21947007

[B25] Niwa M. Daimon Y. Kurotani K. Higo A. Pruneda-Paz J. L. Breton G. (2013). BRANCHED1 interacts with *FLOWERING LOCUS T* to repress the floral transition of the axillary meristems in Arabidopsis. Plant Cell 25, 1228–1242 10.1105/tpc.112.109090 23613197 PMC3663264

[B26] Pin P. A. Nilsson O. (2012). The multifaceted roles of *FLOWERING LOCUS T* in plant development. Plant Cell Environ. 35, 1742–1755 10.1111/j.1365-3040.2012.02558.x 22697796

[B27] Schmid M. Davison T. S. Henz S. R. Pape U. J. Demar M. Vingron M. (2005). A gene expression map of *Arabidopsis thaliana* development. Nat. Genet. 37, 501–506 10.1038/ng1543 15806101

[B28] Searle I. He Y. Turck F. Vincent C. Fornara F. Krober S. (2006). The transcription factor FLC confers a flowering response to vernalization by repressing meristem competence and systemic signaling in Arabidopsis. Genes Dev. 20, 898–912 10.1101/gad.373506 16600915 PMC1472290

[B29] Suarez-Lopez P. Wheatley K. Robson F. Onouchi H. Valverde F. Coupland G. (2001). CONSTANS mediates between the circadian clock and the control of flowering in Arabidopsis. Nature 410, 1116–1120 10.1038/35074138 11323677

[B30] Tiwari S. B. Shen Y. Chang H. C. Hou Y. Harris A. Ma S. F. (2010). The flowering time regulator CONSTANS is recruited to the *FLOWERING LOCUS T* promoter via a unique cis-element. New Phytol. 187, 57–66 10.1111/j.1469-8137.2010.03251.x 20406410

[B31] Torti S. Fornara F. Vincent C. Andres F. Nordstrom K. Gobel U. (2012). Analysis of the Arabidopsis shoot meristem transcriptome during floral transition identifies distinct regulatory patterns and a leucine-rich repeat protein that promotes flowering. Plant Cell 24, 444–462 10.1105/tpc.111.092791 22319055 PMC3315226

[B32] Valverde F. Mouradov A. Soppe W. Ravenscroft D. Samach A. Coupland G. (2004). Photoreceptor regulation of CONSTANS protein in photoperiodic flowering. Science 303, 1003–1006 10.1126/science.1091761 14963328

[B33] Wenkel S. Turck F. Singer K. Gissot L. Le Gourrierec J. Samach A. (2006). CONSTANS and the CCAAT box binding complex share a functionally important domain and interact to regulate flowering of Arabidopsis. Plant Cell 18, 2971–2984 10.1105/tpc.106.043299 17138697 PMC1693937

[B34] Yanovsky M. J. Kay S. A. (2002). Molecular basis of seasonal time measurement in Arabidopsis. Nature 419, 308–312 10.1038/nature00996 12239570

[B35] Yoo S. K. Chung K. S. Kim J. Lee J. H. Hong S. M. Yoo S. J. (2005). CONSTANS activates SUPPRESSOR OF OVEREXPRESSION OF CONSTANS 1 through *FLOWERING LOCUS T* to promote flowering in Arabidopsis. Plant Physiol. 139, 770–778 10.1104/pp.105.066928 16183837 PMC1255994

