# Supplementary material for: Post-fertilization expression of FLOWERING LOCUS T suppresses reproductive reversion
Source: Front Plant Sci. 2014 Apr 30;5:164. doi: 10.3389/fpls.2014.00164 (PMC4012189; doi:10.3389/fpls.2014.00164)
Supplement: Supplementary file 1 [file Presentation1.PDF]

Supplemental Figures

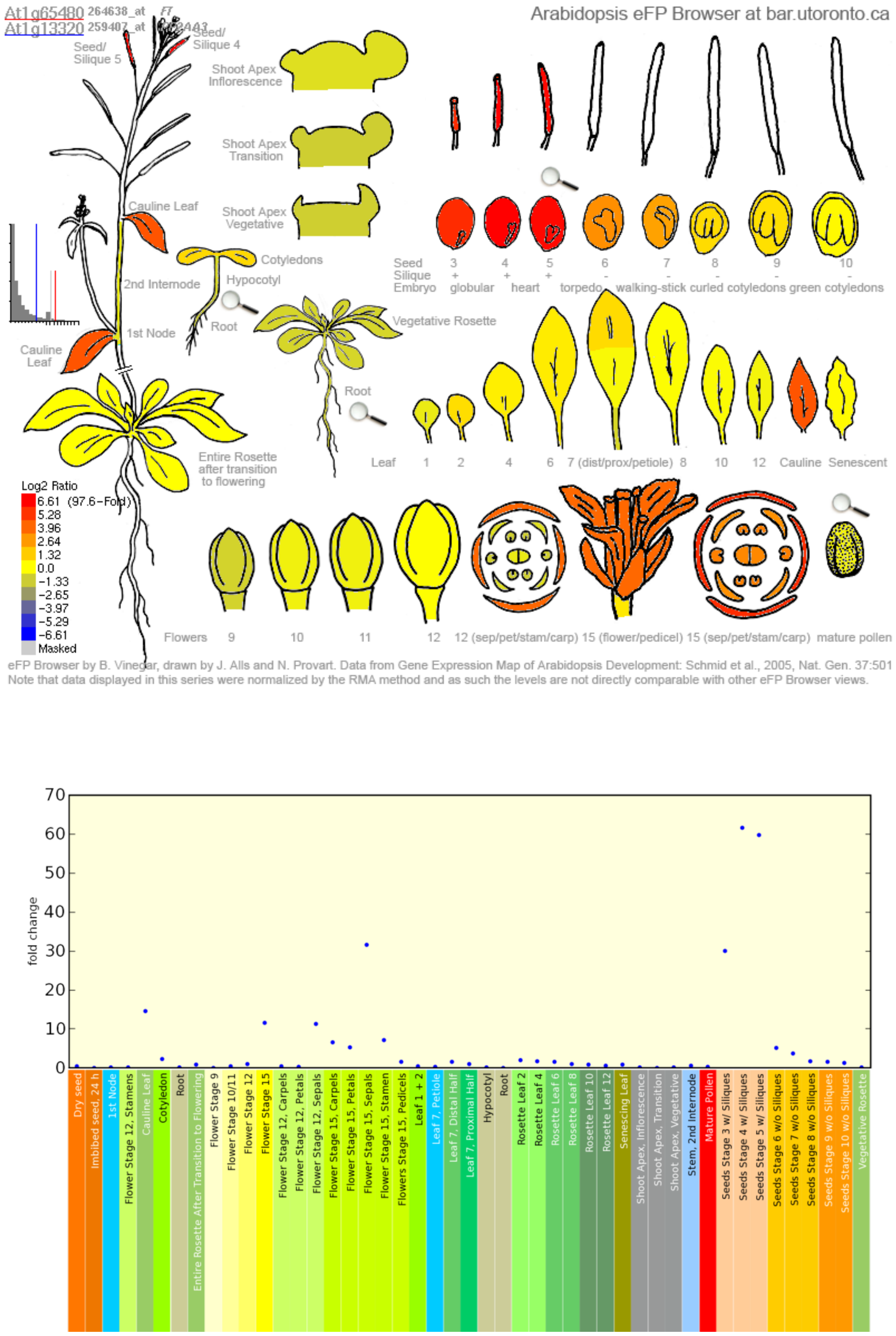

S1. *FT* expression profiles in the life cycle of Arabidopsis based on microarray data. Data were visualized with the eFP browser website of the University of Toronto (http://bar.utoronto.ca/efp/cgi-bin/efpWeb.cgi).

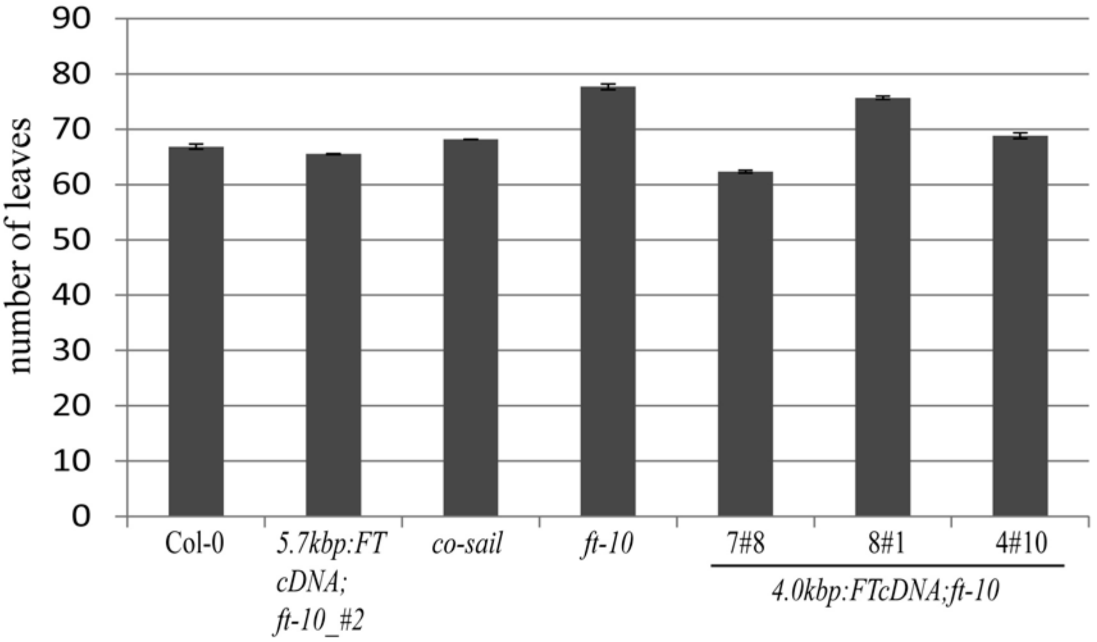

S2. Flowering time of transgenic lines in SDs

Total rosette and cauline leaves of Col-0, *co-sail*, *ft-10* and *ft-10* plants carrying constructs to drive *FT* cDNA by *5.7kbFTp* and *4.0kbFTp* were counted under SD conditions. Numbers of total leaves are shown as the mean ± SE.

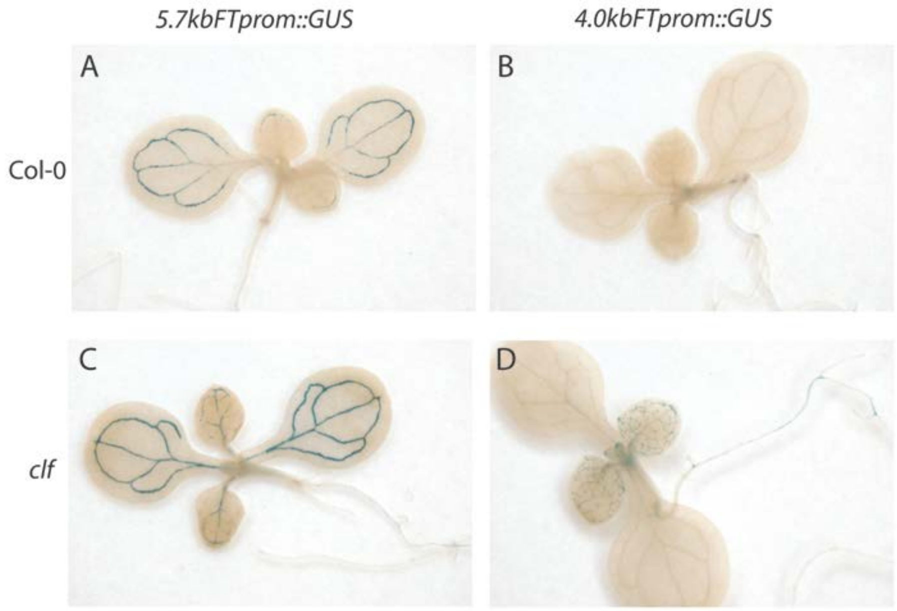

S3. Expression of *FT* promoter GUS constructs in *clf* mutants. Histochemical detection of *GUS* expression in transgenic Col-0 (**A,B**) and *clf* mutant (**C**,**D**) plants under the control of the 5.7 kb *FT* promoter (**A**, **C**) or the 4.0 kb *FT* promoter (**B**, **D**). Seedlings were grown for 8 days on GM medium in LD.
